# ERP44 inhibits human lung cancer cell migration mainly via IP3R2

**DOI:** 10.18632/aging.100984

**Published:** 2016-06-23

**Authors:** Xue Huang, Meng Jin, Ying-Xiao Chen, Jun Wang, Kui Zhai, Yan Chang, Qi Yuan, Kai-Tai Yao, Guangju Ji

**Affiliations:** ^1^ Cancer Research Institute of Southern Medical University, Guangzhou, China; ^2^ National Laboratory of Biomacromolecules, Institute of Biophysics, Chinese Academy of Sciences, Beijing, China; ^3^ Current address: Department of molecular & medical genetics, Oregon Health & Science University, Portland, OR 97239, USA

**Keywords:** calcium, migration, A549, ERP44, IP3Rs

## Abstract

Cancer cell migration is involved in tumour metastasis. However, the relationship between calcium signalling and cancer migration is not well elucidated. In this study, we used the human lung adenocarcinoma A549 cell line to examine the role of endoplasmic reticulum protein 44 (ERP44), which has been reported to regulate calcium release inside of the endoplasmic reticulum (ER), in cell migration. We found that the inositol 1,4,5-trisphosphate receptors (IP3Rs/ITPRs) inhibitor 2-APB significantly inhibited A549 cell migration by inhibiting cell polarization and pseudopodium protrusion, which suggests that Ca2+ is necessary for A549 cell migration. Similarly, the overexpression of ERP44 reduced intracellular Ca2+ release via IP3Rs, altered cell morphology and significantly inhibited the migration of A549 cells. These phenomena were primarily dependent on IP3R2 because wound healing in A549 cells with IP3R2 rather than IP3R1 or IP3R3 siRNA was markedly inhibited. Moreover, the overexpression of ERP44 did not affect the migration of the human neuroblastoma cell line SH-SY5Y, which mainly expresses IP3R1. Based on the above observations, we conclude that ERP44 regulates A549 cell migration mainly via an IP3R2-dependent pathway.

## INTRODUCTION

Invasion and metastasis are common phenomena in cancer cells. Metastasis is generally defined as the spread of malignant cells from the primary tumour through the circulation to establish secondary growth in a distant organ [1]. In most cases, metastasis, not the primary tumour per se, is the main cause of mortality in cancer patients [2]. The incidence of lung cancer is one of the highest worldwide among all cancers [3]. Because of the difficulty of early diagnosis, high transfer and high recurrence rate, lung cancer leads to high fatality. The A549 cell line, which is derived from the tissues of patients with non-small cell lung cancer, has similar biological characteristics as cancer cells within the patient's body. Thus, A549 cells are considered a good research model with which to study the pathogenesis of lung cancer cells.

Accumulating evidence indicates that intracellular Ca^2+^ release and cytosol Ca^2+^ homeostasis are crucial regulators of cell migration [4, 5]. However, if Ca^2+^ participates in the migration of A549 cells, how does Ca^2+^ release regulate A549 cell migration and what is the relationship between Ca^2+^ release and the migration of A549 cells?

ERP44, a pH-regulated chaperon [6], was initially identified as an ER resident protein of the thioredoxin family in co-immunoprecipitation studies with ERO1A in HeLa cells [7]. It was previously reported that ERP44 exhibits its chaperon activity by the retention of formylglycine-generating enzyme in the ER [8] and facilitates the maturation of serotonin transporter [9], IgM [10-12] and adiponectin [13, 14]. Higo and his colleagues [15] reported that ERP44 binds to IP_3_R1, a vital Ca^2+^ channel on the ER membrane, and regulates intracellular Ca^2+^ concentrations by modulating IP_3_R1 activity. Our previous studies showed that IP_3_-induced Ca^2+^ release is decreased in ERP44 overexpressed HeLa cells and that C160/212 of ERP44 influences the binding capacity between IP_3_R1 and ERP44 [16]. However, the role and mechanism of ERP44 in regulating cell migration are unknown. In the present study, we have investigated the role of ERP44 over-expression in the regulation of A549 cell migration. We demonstrate that ERP44 overexpression inhibits A549 cell migration by regulating intracellular Ca^2+^ release from ER Ca^2+^ stores and the inhibitory effect of ERP44 overexpression on A549 cell migration is mainly dependent of IP_3_R2.

## RESULTS

### Observation of Ca^2+^ signalling in A549 cancer cells

**Figure 1:**
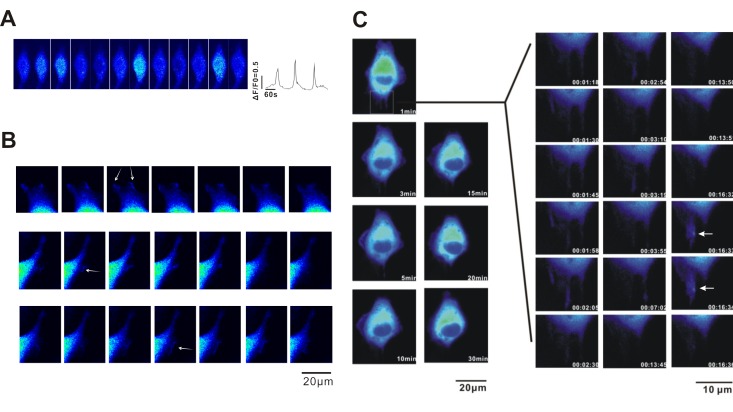
Observation of spontaneous Ca^2+^ transient in A549 cells A549 cells were loaded with fluo-4 AM, and Ca^2+^ activities were recorded using confocal microscopy. (**A**) Ca2+ transients recorded in the xy-t mode. (**B**) No calcium signalling was observed in the leading area with relatively low calcium concentration. The arrows indicate ruffling pseudopodium. (**C**) Long-term (120 min) recording of A549 cell migration in A549 cells detected using the calcium indicator protein GCaMP J. The scanning images were recorded using an API live cell imaging system with pseudo-colour treatment (60× objective lens, 512×512 pixels, XYT scanning, 5 s/frame).

To determine whether A549 cancer cell migration is regulated by intracellular Ca^2+^ signalling, we first detected Ca^2+^ release events using confocal microscopy. A549 cells were loaded with Fluo-AM and confocal microscopy scanning images were collected (40×objective lens, 512×512 pixel, xy-t scan, 1 s/frame without intervals). As shown in Fig. 1A, spontaneous calcium transients were detected in A549 cells. However, no calcium signalling was found in the leading area (Fig.1B, as indicated by arrows) with a relatively low calcium concentration as reported in fibroblasts [4]. To determine the relationship between Ca^2+^ signalling and A549 cell migration, we constructed a calcium indicator protein GCaMPJ [17] that was stably expressed in the A549 cell line. A long-term (120 min) recording of A549 /GCaMPJ cell migration and the scanning images of Ca^2+^ signalling were recorded using the API live cell imaging system (60×objective lens, 512×512 pixels, xyt scanning, 5 s/frame). Spontaneous calcium transients were observed in the cells (Fig.1C). However, we did not identify any relationship between cell migration and the localization of Ca^2+^ signalling in A549 cells.

### Role of Ca^2+^ signalling in A549 cell migration

**Figure 2:**
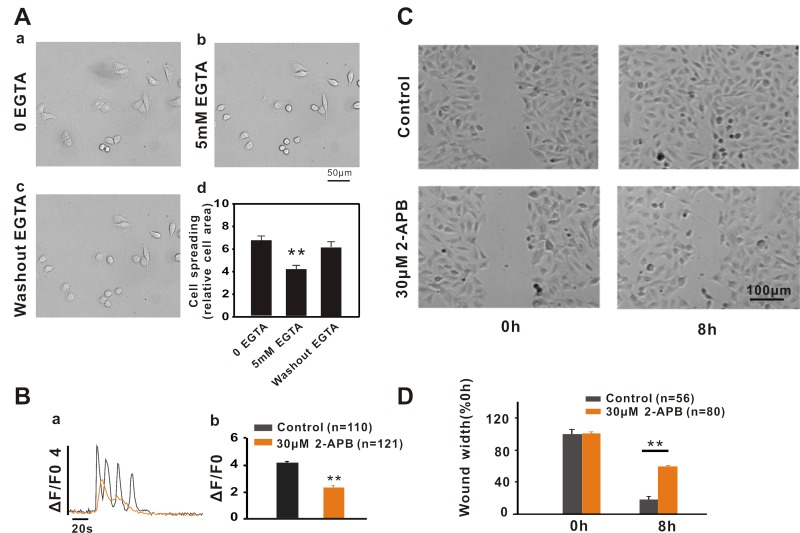
Ca^2+^ signalling regulates A549 cell migration by affecting cell adhesion (**A**) The DIC images show changes in cell adherence during the time lapse under different conditions. (b) 5 mM EGTA in extracellular solution inhibited A549 cell adhesion compared to the control (a) and the inhibitory effect of EGTA on cell adhesion was abolished by EGTA washout (c). The bar graphs (d) show the relative cell area calculated for statistical analysis. (**B**) The IP3Rs inhibitor 2-APB inhibits A549 cell migration via the inhibition of Ca^2+^ signalling. 30 μM 2-APB inhibited ATP-induced calcium release via IP3Rs (a and b) and significantly inhibited wound healing in A549 cells (**C** and **D**).

As noted above, Ca^2+^ events were observed in the lung cancer cell line A549, and the properties of Ca^2+^ release in A549 cells are different from those reported in fibroblasts [4]. These findings motivated further exploration of the role of Ca^2+^ signalling in the regulation of A549 cell migration. It has been previously reported that cell adhesion plays an important role in cell migration [18]. Thus, we first investigated whether extracellular Ca^2+^ buffered with EGTA affects A549 cell adhesion. As shown in Fig. 2A, time-lapse recordings revealed that A549 cell adherence was altered under different conditions. Moreover, 5 mM EGTA (Fig. 2Ab) in the extracellular solution significantly inhibited A549 cell adhesion compared to the control (Fig. 2Aa) and the inhibitory effect of EGTA on A549 cell adhesion was abolished by EGTA washout (Fig. 2Ac). In the next series of experiments, we examined the effects of intracellular Ca^2+^ release on A549 cell migration. First, we investigated the effect of the IP_3_Rs inhibitor 2-APB on Ca^2+^ release induced by ATP in A549 cells. Compared to the control, the peak of Ca^2+^ transients was remarkably reduced in response to the application of 30 μM 2-APB (Fig. 2B, p < 0.01, n = 121 cells). Similarly, in the presence of 2-APB (30 μM), the wound healing of A549 cells was greatly retarded (Fig. 2C & D), which suggests that Ca^2+^ release plays a role in regulating A549 cell migration and that the inhibition of Ca^2+^ release delays A549 cell migration. Next, to determine whether ryanodine receptor Ca^2+^ release channels play the same role as IP3Rs in regulating A549 cell migration, wound healing studies were performed. The results indicated that A549 cell migration was not affected by the application of 20 μM ryanodine (supplementary Fig. S1), suggesting that IP_3_Rs, but not RYRs, are involved in regulating the migration of A549 cancer cells.

### 2-APB inhibits A549 cell migration via altering cell polarization

**Figure 3:**
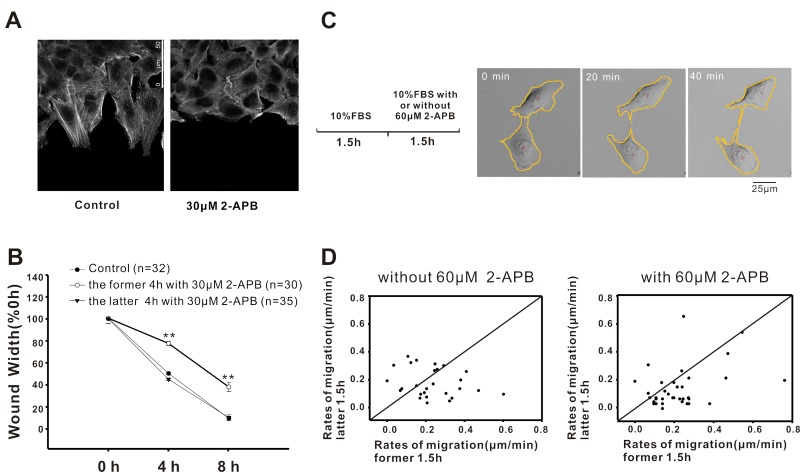
Inhibition of Ca^2+^ signalling inhibits A549 cell migration by affecting cell polarization (**A**) Images show that the polarization and pseudopodium protrusion of A549 cells were inhibited in the presence of 2-APB (30 μM). (**B**) Treatment with 2-APB during the latter half of the assay did not affect wound healing. (**C**) Real-time recording of A549 cells' random motility. The sequential pictures were shown to explain the computing method. The centre of gravity was calculated based on the outlines of the cells (red circle), and the change in the coordination of the centre represented the tracking of cell movement. (**D**) Statistical analysis of the migration rate for each cell. The x-axis represents the cell rates during the initial 1.5 h, and the y-axis represents the cell rates during the latter 1.5 h. The dots in the lower right diagonal represent the cells with decreased rates.

The results described above suggest that the inhibition of intracellular Ca^2+^ release by 2-APB resulted in retarded wound healing in A549 cells. In the next series of experiments, we investigated how inhibiting the release of intracellular Ca^2+^ retarded A549 cell migration. Cell polarization is the basis for cell migration [19]. Therefore, we first examined the effect of Ca^2+^ release inhibition on cell polarization by staining A549 cells with Phalloidin-FITC. As shown in Fig. 3A, stimulation of the scratch resulted in a trend in cell polarization. More pseudopodium protrusion and stress fibers were observed within 2 h after scratching in A549 cells. In contrast, in the presence of 2-APB (30 μM), the polarization and pseudopodium protrusion of A549 cells were significantly inhibited, suggesting that 2-APB inhibited A549 cell migration by affecting cell polarization. Is this the mechanism by which 2-APB inhibits A549 cell migration? To address this question, we exposed cells to 2-APB at different time periods in cell scratch experiments. Cells treated with 2-APB during the first 4 h showed a significant inhibition of wound healing; conversely, cells treated with 2-APB in the latter 4 h did not affect cell wound healing (Fig. 3B), which confirmed that 2-APB inhibited A549 cell migration by inhibiting cell polarization.

Next, we examined the effect of 2-APB on A549 cell random motility. As shown in Fig. 3C, during the first 1.5 h, the cells were treated with 10% FBS only; in the latter 1.5 h, cells were treated without or with 60 μM 2-APB except for 10% FBS. The centre of gravity was calculated based on the outlines of the cells (red circle), and the change in the coordination of the centre represented the tracking of cell movement. Figure 3D shows the migration rate for each individual cell. Compared to the control (left panel, 15/27), the number of cells with declining migratory rate was increased when the cells were treated with 60 μM 2-APB (right panel, 29/38) during the latter 1.5 h, indicating that 2-APB decreased the migration of A549. Taken together, these results indicated that the IP_3_R inhibitor 2-APB inhibited A549 cancer cell migration by affecting cell polarization by reducing intracellular Ca^2+^ release.

### ERP44 overexpression inhibits A549 cell migration by inhibiting intracellular Ca^2+^ release

**Figure 4:**
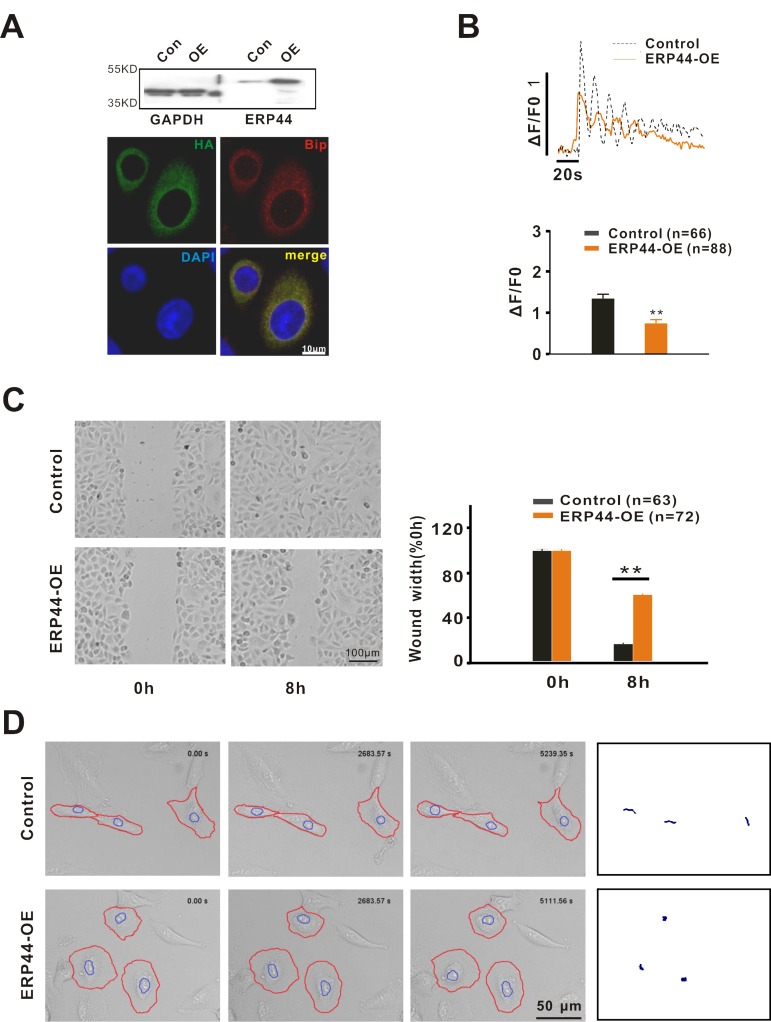
ERP44 inhibits cell migration by reducing intracellular Ca^2+^ release (**A**) Identification of ERP44 overexpression (ERP44-OE) system in A549 cells via western blot and immunofluorescence. Overexpressed ERP44 were co-located with ER marker Bip. (**B**) ERP44 overexpression inhibited 10 μM ATP-induced calcium release via IP3Rs. (**C**) Wound healing was significantly inhibited by overexpressed ERP44. (**D**) Overexpression of ERP44 inhibited A549 cells random motility. A549 cells were recorded in real time after adenovirus infection. Circled cells are DsRed-positive cells. The right panel shows the movement tracking of A549 cells.

ERP44 is a member of the PDI family and was the first protein observed to bind to and exhibit an inhibitory effect on the IP_3_R1 Ca^2+^ release channel inside of the ER [15]. Based on our observation that 2-APB inhibited the migration of A549 cells, we postulated that the inhibitory effect of 2-APB on A549 cell migration was mediated by IP_3_Rs. Thus, in the next series of experiments we examined the role of ERP44 overexpression in A549 cells. Due to the low efficiency of the transient transfection of pCMV-*ERP44*-IRES-DsRed2 in A549 cells (<5%), adenovirus infection was used to over-express ERP44 in the cells. As shown in Fig. 4A, with the adenovirus infection approach ERP44 was highly expressed in ER of A549 cells. Compared to control A549 cells (DsRed2 only), ERP44 overexpression significantly inhibited ATP-induced Ca^2+^ release (Fig. 4B). Further studies indicated that the overexpression of ERP44 remarkably inhibited wound healing in A549 cells (Fig. 4C). Long-term real-time tracking analysis of cell movement indicated that the overexpression of ERP44 inhibited A549 cell random motility, and similar to the effect of 2-APB on A549 cells, the cells exhibited a “fried egg” morphology, indicating that the polarization and pseudopodium protrusion of A549 cells were inhibited by the over-expression of *ERP44* (Fig. 4D). The physical centre of gravity in ERP44 overexpressed A549 cells was nearly maintained at its original location during the 1.5 h tracking time.

As we noted above, 2-APB inhibited Ca^2+^ release and resulted in an inhibitory effect on A549 cell migration by affecting the cell cytoskeleton. Thus, we examined whether ERP44, similar to 2-APB, also inhibited cell migration by affecting the cell cytoskeleton. In the control, A549 cells stained with Phalloidin-FITC exhibited a clear structure consisting of F-actin microfilaments (Supplementary Fig. 2) and polarized cells presented a network arrangement of microfilaments at the forefront of the cells. In addition, stress fibres were observed throughout the cells. However, the microfilaments were not clearly observed or only some circular microfilaments were observed around the edge of the cells in ERP44 overexpressed A549 cells, suggesting that ERP44, similar to 2-APB, inhibited A549 cell migration by affecting the cell cytoskeleton.

### ERP44 inhibition of A549 cell migration is mainly dependent on IP_3_R2

**Figure 5:**
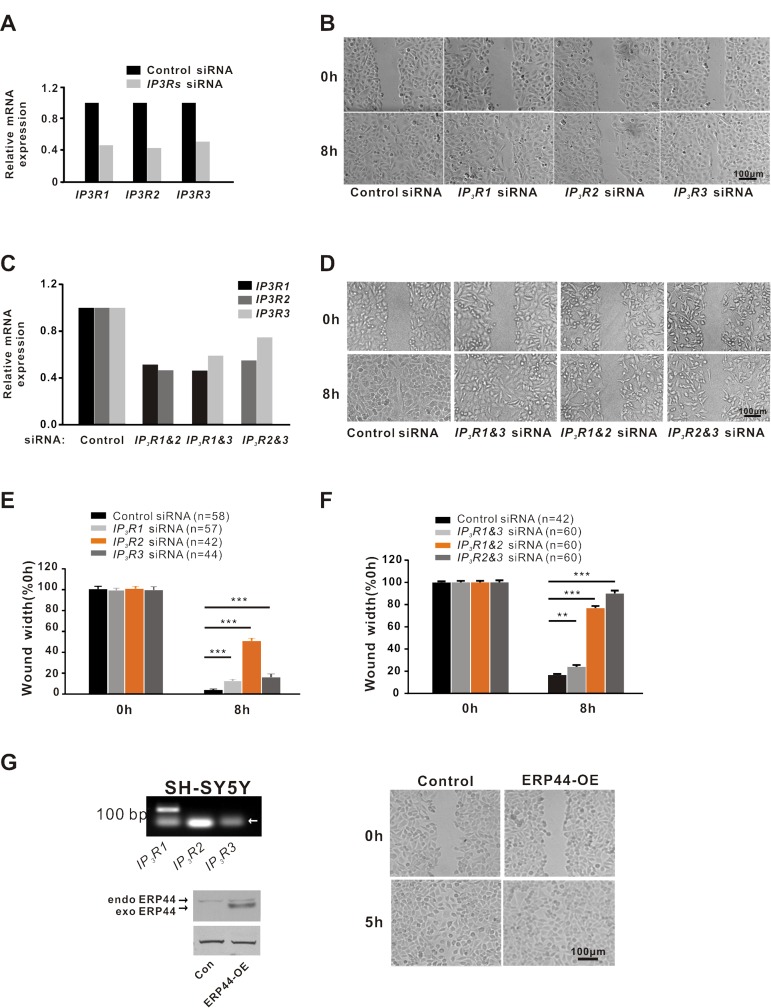
IP3R2 plays a dominant role in regulating A549 cell migration (**A**) RT-PCR analysis for the three subtypes of *IP3Rs* expression in A549 cells with control or single *IP3Rs* siRNA. (**B)** Wound healing in A549 cells with control or single *IP3Rs* siRNA. (**C**) The interference efficiency detection in A549 cells with control or combined *IP3Rs* siRNA. (**D**) Wound healing in A549 cells with control or combined *IP3Rs* siRNA. (**E**) Statistical analysis of single *IP3Rs* siRNA affecting wound healing in A549 cells. (**F**) Statistical analysis of combined *IP3Rs* siRNA affecting wound healing in A549 cells. (**G**) Overexpression of ERP44 did not affect wound healing in SH-SY5Y cells. RT-PCR assay shows that IP3R1 is specifically expressed in SH-SY5Y cells (left-upper). The arrow indicates primer dimers. Western blotting detection of ERP44 in SH-SY5Y transfected with control or *ERP44* overexpression adenovirus (left-lower). The arrows indicate endogenous and exogenous ERP44. Overexpression of ERP44 did not affect wound healing in SH-SY5Y cells (right).

It has been reported that ERP44 inhibits intracellular Ca^2+^ release by binding to IP_3_R1 [15]. We confirmed that all three types of IP_3_R were expressed in A549 cells (Fig. 5A). However, the subtype of IP_3_Rs that mediates the inhibitory effect of ERP44 on A549 cell migration remains unknown. To clarify this, we performed RNA interference studies. We synthesized siRNAs for *IP_3_R1*, *IP_3_R2* and *IP_3_R3* according to a previously reported method [4] and the real-time PCR results indicated the interference efficiency of single *IP_3_Rs* siRNA to be >50% after transfection for 72 h (Fig. 5A). Wound-healing studies demonstrated that all types of IP_3_Rs exhibited a inhibition of wound healing of A549 cells compared to the control (Fig. 5B & E, p < 0.001 vs. control). However, among these receptors, IP_3_R2 displayed a remarkable inhibitory effect on A549 cell wound healing (Fig. 5B & E, p < 0.001 vs. IP_3_R1 and IP_3_R3). To further confirm, we carried out wound-healing studies with combined *IP_3_Rs* siRNA of >30% interference efficiency. As the Fig. 5D & F shown, wound healing in A549 cells with treatment involved *IP_3_R2* siRNA was markedly inhibited while in A549 cells with *IP_3_R1* and *IP_3_R3* siRNA was mildly inhibited. These results suggested that IP_3_R2 plays a predominant role in mediating the inhibitory effect of ERP44 on A549 cell migration. Moreover, we performed scratch experiments in ERP44 stably transfected SH-SY5Y cells, which mainly express IP_3_R1 [20](Fig. 5G left-upper), indicated that the overexpression of ERP44 did not significantly inhibit cell migration, confirming that ERP44 inhibition of cell migration is independent of IP_3_R1 (Fig. 5).

## DISCUSSION

During the past two decades, several studies have reported that calcium is involved in the regulation of cell migration. Calcium plays a crucial role in the cytoskeletal organization by its interplay with filamin A, α-actinin, gelsolin, villin, scinderin, severin or calcineurin B [21-24]. The treadmilling process of actin is strictly controlled by calcium. The activation of the myosin light chain kinase and its regulatory kinase CaMKII, which are implicated in cell contraction, is dependent on the cytoplasmic concentration of calcium [25]. The calpain family is a type of calcium-dependent proteolytic enzyme responsible for the dissociation of cell adhesion [26]. Moreover, calcium signalling regulates the activation and translocation of Rac, which is implicated in the protrusion of lamellipodia and tumour progression [27].

To date, there are two approaches to studying the relationship between calcium and cell migration. The first approach is to record the calcium signalling in real time to reveal the mechanism of cell migration. Calcium gradients are found in polarized eosinophils [28] followed by the observation that calcium transients increases in mobile epithelial keratocytes [29] and calcium oscillations regulate thymocyte motility [30]. Another indirect approach to studying the role of calcium in cell migration is to modulate the intracellular calcium using RNA interference or treatment with inhibitors of calcium channels. The blockade of type 3 ryanodine receptors with ryanodine strongly attenuated astrocyte motility [31]. A reduction in ORAI1 or STIM1, both of which are essential for store-operated calcium entry, in breast tumour cells decreased tumour metastasis [32]. In addition, the migratory potential of nasopharyngeal carcinoma cells is decreased by extracellular Ca^2+^ chelators, TRPM7 inhibitors or TRPM7 knockdown [33].

In the present study, we have combined the two methods described above to examine the effect of intracellular calcium activities on A549 cell migration. We observed spontaneous Ca^2+^ signalling in A549 cells, which confirmed that there are spontaneous calcium activities in non-excitable cells followed by fibroblasts [4], although determining the correlation between spontaneous calcium activities and A549 migration will require further study. Moreover, we found that intracellular Ca^2+^ release via IP_3_Rs played an important role in the regulation of A549 cell migration by treatment with the inhibitor 2-APB, overexpression of inhibitory protein ERP44 or RNA interference of IP_3_Rs. The inhibitor of IP_3_Rs, 2-APB, has long been used, although it is not as selective as xestospongin C. In the presence of 2-APB, the migration of A549 cells was significantly inhibited (Fig. 2) and the inhibition of 2-APB on A549 cell migration was mediated by a reduction in the intracellular Ca^2+^. Moreover, the inhibitory effect of 2-APB on cancer cell migration is achieved by the inhibition of cell polarization and pseudopodium protrusion of A549 cells.

It has been reported that ERP44 inhibits intracellular Ca^2+^ release by binding to IP_3_R1 inside of ER [15]. Based on this effect of ERP44 on Ca^2+^, we speculate that ERP44 is involved in cancer cell migration. Indeed, the overexpression of ERP44 inhibited intracellular Ca^2+^ release followed by significantly retarded A549 cell migration (Fig. 4). As with 2-APB, the inhibitory effect of ERP44 on A549 cell migration was achieved by altering the polarization and pseudopodium protrusion of A549 cells. Similarly, IP_3_Rs knockdown experiments resulted in the inhibition of A549 cell migration, and IP_3_R2 displayed an unexpected significance in inhibiting A549 cell migration, which indicates that IP_3_R2 plays the most important role in regulating A549 cancer cell migration. This finding was confirmed by the observation that the stable overexpression of ERP44 did not affect SH-SY5Y cell migration (Fig. 5).

On the basis of the findings above, we conclude that intracellular Ca^2+^ release via IP_3_Rs plays an important role in the regulation of A549 cell migration and the Ca^2+^ release of A549 cells is regulated by ERP44 mainly via IP_3_R2.

## METHODS

### Cell culture

A549 cells and SH-SY5Y cells were cultured in RPMI 1640 medium supplemented with 10% foetal bovine serum and 100 U/mL penicillin and streptomycin. AD-293 cells were cultured in Dulbecco's modified Eagle's medium supplemented with 10% foetal bovine serum and 100 U/mL penicillin and streptomycin. Foetal bovine serum (GIBCO) was inactivated at 56°C for 30 min. All the cell lines purchased from the Cell Bank of Type Culture Collection of the Chinese Academy of Sciences (Shanghai, China) and have been tested and authenticated utilizing Short Tandem Repeat (STR) profiling.

### Adenoviral(Ad) overexpression system

ERP44 overexpression was achieved by recombinant adenoviral infection. Plasmids for pCMV-IRES-DsRed and pCMV-*ERP44*-IRES-DsRed were first obtained. Next, recombinant Ad plasmids and Ad packaging were constructed using the AdEasy™ XL Adenoviral Vector System™ (Stratagene).

### RNA interference

siRNA duplexes were purchased from GenePharma. The target sequences were siControl (5′-AAGUAGUGUAUGCUAGAGUGG-3′), si*IP_3_R1* (5′-GGCCUGAGAGUUACGUGGCAGAAAU-3′), si*IP_3_R2* (5′-GAGAAGGCUCGAUGCUGAGACUUGA-3′), and si*IP_3_R3* (5′-CCUACCUGCUGUCUGUCUU-3′). Cells were transfected using siRNA by Lipofectamine 2000 (Invitrogen).

### Western blotting assay

Proteins were resolved by SDS-PAGE and transferred onto a polyvinylidene difluoride membrane (Millipore). The membranes were blocked with 5% skim milk in TBS containing 0.1% Tween-20 (TBST) for 1 h and probed with the primary antibody at 4°C overnight. The antibodies used in this study were as follows: ERP44 rabbit monoclonal antibody (Cell Signaling) and GAPDH antibody (Sigma). After being washed with TBST, the membranes were incubated with the appropriate secondary antibody and the signals were detected using an ECL kit (Millipore) or BCIP/NBT kit (Beyotime).

### RT-PCR

Total RNAs were isolated by TRIzol (Invitrogen) and cDNA was prepared with PrimeScript Reverse Transcriptase (Takara). Real-time PCR was performed using the SYBR PrimeScript RT-PCR Kit II (Takara). The following primers were used: *18sRNA* (F: 5′-GGAAGGGCACCACCAGGAGT-3′, R: 5′-TGCAGCCCCGGACATCTAAG-3′), *GAPDH* (F: 5′-GATTCCACCCATGGCAAATTC-3′, R: 5′-AGCATCGCCCCACTTGATT-3′), *IP_3_R1* (F:5′-CCACAGACGCAGTGCTACTCA-3′, R: 5′-TTGCCATACTGGATTACGGT-3′), *IP_3_R2* (F: 5′-AACCTCATGGCAAAGCTATCA-3′, R: 5′-CGAACAGGCACCACGGAC-3′), *IP_3_R3* (F: 5′-CCTGGAAATCCTTCAGTTCAT-3′, R:5′-CATGTTGGCAGTGGTAGAGTC-3′).

### Wound-healing assay

Cells were allowed to form an 85% confluent monolayer in a 35-mm dish coated with 0.1% gelatine prior to wounding. A549 cells were typically seeded at 3-5×10^5^ per dish. Prior to wounding, the cells were starved in serum-free medium for 12-24 h until the cells were 100% confluent. Next, a 10-20 μL pipette tip was used to generate a wound, which was vertical to the parallel line (the interval was 2-3 mm) marked on the bottom of the dish. Typically, it took 6-8 h for the wounds of A549 and SH-SY5Y cells to close. Images were obtained using confocal microscopy with 10× objective lenses based on markers on the bottoms of the dishes, which allowed for a comparison of each of the images at different time points. The wound width and area were analysed using Digimizer.

### Time-lapse cell migration recording

The random migration of A549 cells was recorded using two systems. The differential interference contrast (DIC) image of cells seeded in a 35-mm dish was scanned with a 10×confocal microscopic objective (Leica) for up to 1.5 h of recording with 2-min time intervals. For calcium imaging during cell migration, the API Delta vision live cell working station (API) was used. A549 cells transfected with the GCaMPJ adenovirus were seeded in a 35 mm dish with a glass bottom. After cell adhesion, long-term recording was fulfilled at 5 s/frame with a 60× objective. Images were analysed using ImageJ and SoftWoRx Explore (API).

### Immunofluorescence assay

The cells plated on coverslips were fixed in 4% PFA for 15 min. After being washed in PBS, the cells were permeabilized with 0.1% Triton X-100 for 15 min and washed again in PBS. Cells were blocked with goat serum (ZSGB-BIO) for 1 h at room temperature. Primary antibodies (HA: CWBIO; Bip: abcam) were incubated overnight at 4°C. Wash three times in PBS. Secondary antibodies (ZSGB-BIO) were incubated for 1 h at room temperature. Wash three times in PBS. F-actin was stained using phalloidin-FITC (Sigma) at 2.5 μg/mL for 1 h, and washed in PBS. Nuclei were stained with 7.5 μM propidium iodide (Sigma) for 5 min. The coverslips were mounted onto slides with anti-fading solution (Beyotime).

### Ca^2+^ imaging

Cells were seeded onto 0.1% gelatine-coated coverslips or 35 mm dishes with a glass bottom. Cells with 2-APB treatment were treated with 30 μM 2-APB for 30 min and then incubated with 0.1-0.2 μM Fluo-4 AM (Invitrogen) at room temperature for 10-15 min. Perfusion with a standard solution (150 mmol/L NaCl, 5.5 mmol/L KCl, 2 mmol/L CaCl_2_, 1 mmol/L MgCl_2_, 10 mmol/L HEPES and 3 mmol/L glucose, pH 7.4). Calcium signalling was scanned (xyt scan and no time interval) using confocal microscopy (Leica) at 488 nm wavelength excitation with a 40× objective. Images were analysed using ImageJ.

### Statistical analysis

Data were expressed as the mean ± s.e.m. from three independent experiments and analysed using t-test with significance defined as ** p < 0.01; ***p < 0.001.

## SUPPLEMENTARY DATA FIGURES


