# Supplementary material for: ERP44 inhibits human lung cancer cell migration mainly via IP3R2
Source: Aging (Albany NY). 2016 Jun 23;8(6):1276–85. doi: 10.18632/aging.100984 (PMC4931832; doi:10.18632/aging.100984)
Supplement: Supplementary file 1 [file aging-08-1276-s001.pdf]

SUPPLEMENTARY DATA

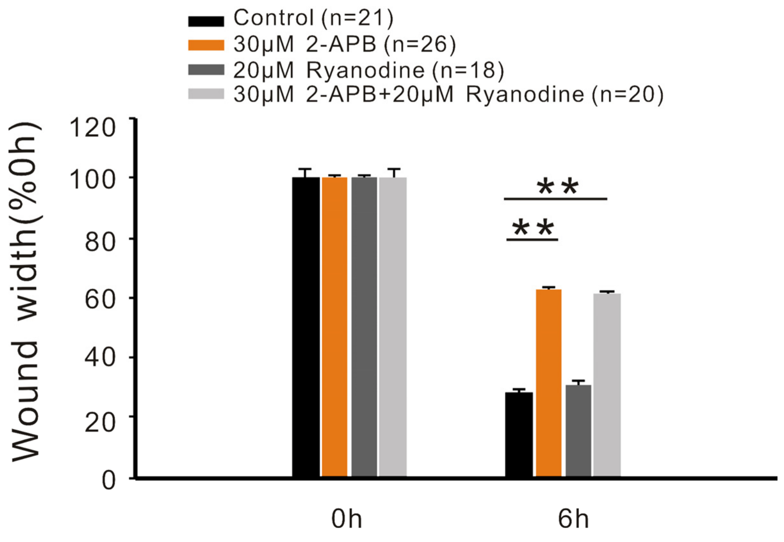

Supplementary Figure 1. IP3Rs but not RYRs are required in A549 cell migration. IP3R inhibitor, 2-APB, and RYRs inhibitor, ryanodine, were used to expore Ca^{2+} release channels involved in A549 cell migration. The wound healing of A549 cells that added 30μM 2-APB was obviously inhibited while that of cells stimulated by 20μM ryanodine was not affected. When the cells added 20μM ryanodine along with 30μM 2-APB, the inhibitory effect was shown again.

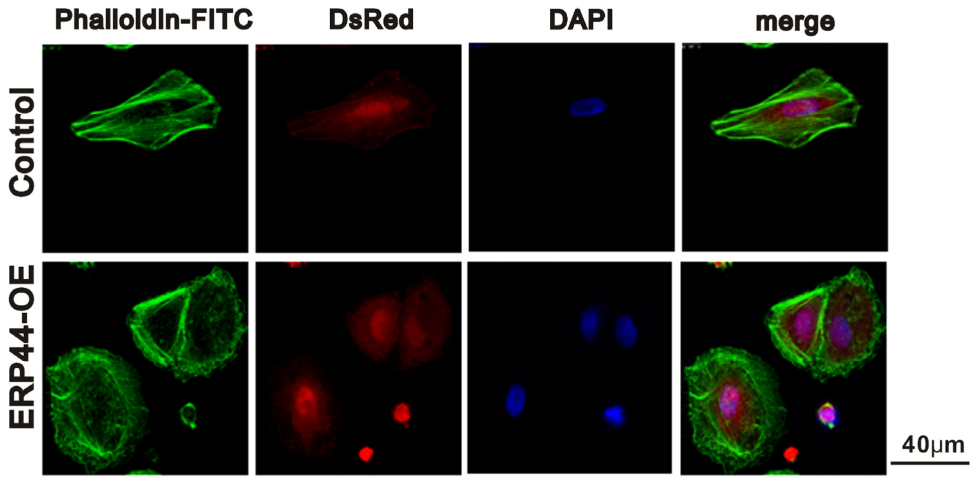

Supplementary Figure 2. ERP44 overexpression leads to change of cell morphology. In control, the A549 cells show polygonal morphology and the microfilaments arrayed regularly while the ERP44 overexpressed cells tend to rounded shape and the microfilaments appear in disorder. Phalloidin-FITC shows the structure of F-actin microfilament; DsRed stands for overexpression level; DAPI indicates nuclei.
